# Mechanical Forces in Floral Development

**DOI:** 10.3390/plants11050661

**Published:** 2022-02-28

**Authors:** Kester Bull–Hereñu, Patricia dos Santos, João Felipe Ginefra Toni, Juliana Hanna Leite El Ottra, Pakkapol Thaowetsuwan, Julius Jeiter, Louis Philippe Ronse De Craene, Akitoshi Iwamoto

**Affiliations:** 1 Fundación Flores, Ministro Carvajal 30, Santiago 7500801, Chile; kester@laboratorioflores.cl; 2 Museo Nacional de Historia Natural, Área Botánica, Parque Quinta Normal S/N, Santiago 8350701, Chile; 3 Centre for Ecology Evolution and Environmental Changes (cE3c), Faculdade de Ciências, Universidade de Lisboa, Campo Grande, Edifício C2, Piso 5, 1749-016 Lisbon, Portugal; papsantos@fc.ul.pt; 4 Department of Environmental Sciences–Botany, University of Basel, Schönbeinstrasse 6, 4056 Basel, Switzerland; 5 Faculty of Biological Sciences, Friederich Schiller University of Jena, 07743 Jena, Germany; ipegtoni@gmail.com; 6 Department of Botany, Institute of Biological Sciences, University of São Paulo, São Paulo 05508-090, Brazil; juliana.ottra@usp.br; 7 Open University of Brazil, Federal University of ABC, Santo André 09210-580, Brazil; 8 Department of Biology, Faculty of Science, Sanam Chandra Palace Campus, Silpakorn University, Nakhorn Pathom 73000, Thailand; thaowetsuwan_p@su.ac.th; 9 Nees-Institute for Biodiversity of Plants, University of Bonn, Meckenheimer Allee 170, 53115 Bonn, Germany; jjeiter@uni-bonn.de; 10 Royal Botanic Gardens Edinburgh, Edinburgh EH3 5LR, UK; lronsedecraene@rbge.org.uk; 11 Department of Biological sciences, Faculty of Science, Kanagawa University, Hiratsuka 259-1293, Japan

**Keywords:** floral development, flower shape, growth forces, mechanical forces, organ imprint, pressure

## Abstract

Mechanical forces acting within the plant body that can mold flower shape throughout development received little attention. The palette of action of these forces ranges from mechanical pressures on organ primordia at the microscopic level up to the twisting of a peduncle that promotes resupination of a flower at the macroscopic level. Here, we argue that without these forces acting during the ontogenetic process, the actual flower phenotype would not be achieved as it is. In this review, we concentrate on mechanical forces that occur at the microscopic level and determine the fate of the flower shape by the physical constraints on meristems at an early stage of development. We thus highlight the generative role of mechanical forces over the floral phenotype and underline our general view of flower development as the sum of interactions of known physiological and genetic processes, together with physical aspects and mechanical events that are entangled towards the shaping of the mature flower.

## 1. Introduction

**Figure 1:**
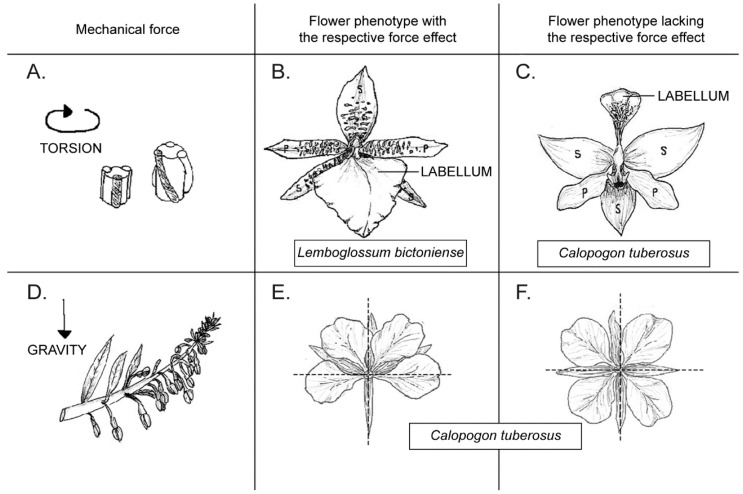
Conceptual proposition of role of mechanical forces on flower phenotype. Pictures in middle (**B**,**E**) show wild phenotype of a given flower when expected force shown to its left (**A**,**D**, respectively) has acted during its ontogeny. Alternatively, images at right (**C**,**F**) show the analogous phenotype when that force is not present during ontogeny, highlighting the generative role of respective mechanical force in creating expected flower phenotype: (**A**) depiction of torsion force taking place in a peduncle of a resupinating orchid. Here, torsion is explained as a derived growing force after peduncle extension (represented by a tube) took place (smaller at left before growth; larger at right after growth). Existing lateral ribs of peduncle, represented by three thinner stripes, enlarge more than central tube, and thus forcedly adopt a spiral display to spatially accommodate extra tissue generated within ribs. Torsion of whole structure shown on right is a consequence of this differential growth. One of the ribs is shaded for illustrative purposes. (**B**) Typical resupinated flower of an orchid: in this example, *Lemboglossum bictoniense,* with its labellum in ventral (abaxial, downwards) position. (**C**) A nonresupinating flower of orchid *Calopogon tuberosus* with its labellum in its original dorsal (adaxial, upwards) position. Note differential position of sepals and petals, and in particular, showy labellum depending on whether torsion force took place (**B**) or not (**C**) during floral development. P = petal, S = sepal. (**D**) Depiction of gravity force vector on a growing inflorescence of *Epilobium angustifolium*. (**E**) Mature flower of *E. angustifolium* showing its typical zygomorphic symmetry. (**F**) Flower of same species but showing radial symmetry when plant was grown in a clinoscope, thus canceling effect of gravity. Note difference in resulting symmetry of flower when force of gravity has acted in (**E**) or not (**F**). Abbreviations: P, petal; S, sepal. ((**A**–**C**) Redrawn from Dines and Bell, 1994; (**D**–**F**) from Vöchting, 1886) [9,11].

There was considerable discussion on whether the development of flowers is regulated solely by a genetic program or by mechanical forces, since Green [1,2] demonstrated that the development of organs may be determined by physical forces acting on the floral meristem (see also [3]). The idea of a self-regulating (automated) system is taking hold, especially in the understanding of the regulation of phyllotaxis (e.g., [4,5]). How can mechanical forces be considered as part of an ontogenetic process in a plant? Seen from a broad perspective, every growth process of a plant organ, and in particular, of a flower, involves the occurrence of mechanical forces at the tissue level such as stretching, compression, torsion, ripping, and so on [6,7]. Something that might sound obvious and spurious in the first place acquires biological interest when it is noticed that for a particular floral phenotype to be expressed or achieved, specific internal mechanical events are required throughout ontogeny (Figure 1). As an example, the mature phenotype of the orchid *Lemboglossum bictoniense *(Figure 1B) involves the torsion of its pedicel in a movement known as resupination that turns the flower for 180° (Figure 1A). This torsion is necessary for the labellum of the orchid to adopt a typically lower abaxial position. When this mechanical event does not occur, as in the orchid *Calopogon tuberosus*, the resulting phenotype looks consequently different, with the labellum being placed on top in the adaxial position ([8], Figure 1C). In analogy, the monosymmetric flower of *Chamaenerion *(*Epilobium*) *angustifolium* (Figure 1E) was shown to interact with the gravitational force that acts during the inflorescence development (Figure 1D), as when the plant was grown in a clinoscope that cancels the action of the force of gravity, then the resulting floral symmetry shifts to become radial ([9]; Figure 1D).

Much of the mechanical forces acting in floral ontogeny are invisible to the naked eye as they occur at an early and microscopic level and are even hidden below layers of tissues within reproductive buds. The generative role that forces alone might have been already shown by experiments. Green [2] demonstrated how leaf primordia can be artificially created de novo by the mechanical bulging of a meristematic tissue or in analogy by reducing its cell wall resistance. Furthermore, Ronse De Craene [10] recently reviewed how shape and size of the floral meristems can condition the phenotypic outcome of the flower, and in particular, when pressures take place within the inflorescence bud. Thus, by a logical extension, any internal mechanical force that can alter the size and shape of a floral meristem is necessarily a determinant of at least some aspect of the floral phenotype.

Accepting the fact that certain mechanical forces can contribute to the determination of floral attributes at defined times in the ontogeny, it appears relevant to identify these and to systematize how they could affect the phenotype in an autonomous and consistent way. The aim of this review is to report on and classify mechanical pressures that will be typically acting both in developing floral meristems and in shaping the floral phenotype. First, we refer to known experiments in forces on meristems; next, we classify different kinds of pressures that take place during floral development; and we finish with an outlook on imaging techniques that will help to study and visualize the influence of mechanical forces in floral development.

## 2. Experiments with Mechanical Forces

Although there are many cases that demonstrated that mechanical forces affect floral development (see Section 2), there are relatively few experimental approaches to demonstrate the effect of mechanical forces. This situation does not reflect the fact that the experiments about mechanical forces are not important, but rather that they received much less attention compared with those of molecular and biochemical approaches to the floral development. In this section, we introduce three experimental methodologies reported in previous studies and present a novel experimental system.

### 2.1. Constraining and Microsurgical Experiment

**Figure 2:**
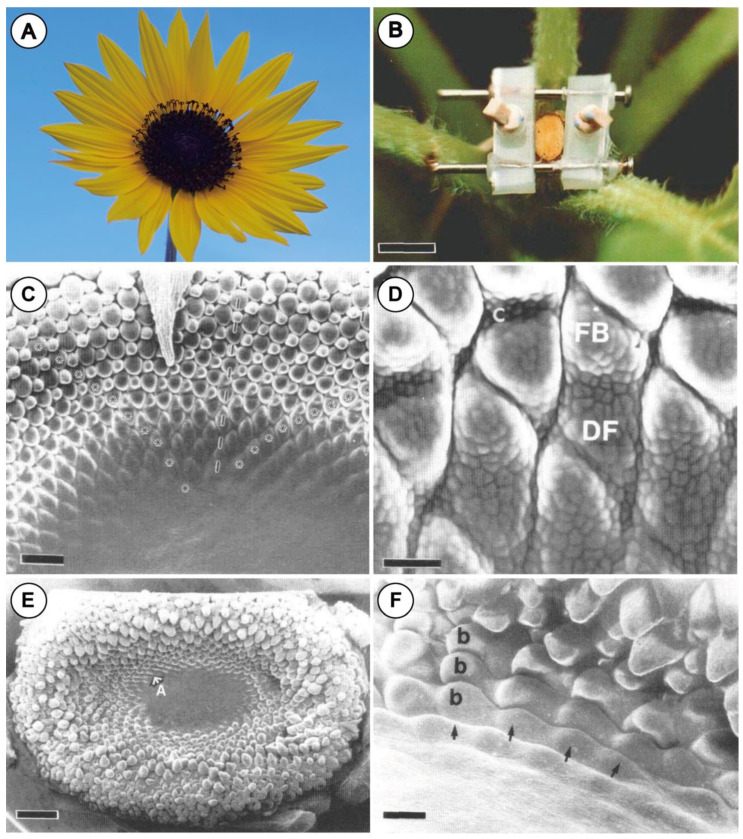
Experiments with mechanical pressures on developing meristems of *Helianthus* after Hernández & Green (1993): (**A**) mature inflorescence of *Helianthus* *annuus.* (**B**) Inflorescence meristem is constrained with device. (**C**) Inflorescence meristem without compression forces by device. Generative zone is an arc-like region. Two spiral parastichies of floret primordia (asterisk) and radius of inflorescence (dashed line) are indicated. (**D**) Developing florets in (**C**). Upper portion of each floret becomes a disc flower bract (FB), and lower portion becomes a disc flower. A transverse crease develops in older florets. (**E**) Inflorescence meristem with 9 days compression by device. “A” denotes area shown in (**F**). (**F**) Developing florets denoted by “A” in (**E**). Floral primordia buckles, which are unusual in that they become single large bracts (b) rather than dyad florets shown in (**D**). Phyllotaxis in floral meristem is also distorted compared with that in a normal meristem in (**C**). Abbreviations: C, crease; DF, disc flower; GZ, generative zone. Scale bar in (**B**) = 5 mm; in (**C**) = 200 µm; in (**D**) = 40 µm; in (**E**) = 1 mm; in (**F**) = 100 µm. ((**B**–**F**) Reproduced from Hernández and Green, 1993 with permission) [12].

Hernández and Green [12] experimented with compression forces on the inflorescence meristem of the sunflower (*Helianthus annuus*; Figure 2A). Here, they laterally constrained the young head of a sunflower conferring an oval shape to it (Figure 2B,E). This resulted in a shift in the phyllotaxis of the floral primordia (Figure 2C,E,F), partly demonstrating the physical buckling theory of Green [1,2]. They also reported that the organ character of floret changed due to the mechanical forces by constraining the young head. The young floret of Sunflower is usually a floral primordium subtended by a bract primordium (a kind of dyad; Figure 2D). Some were, however, replaced by a single large bract (Figure 2F). This experiment demonstrated that the mechanical forces should affect not only the floral configuration in the inflorescence (floral phyllotaxis), but also the development of each flower.

In another study, Dumais and Steele [13] estimated the distribution of compressive stress in the meristematic head of the sunflower with a microsurgical manipulation. They made shallow cuts on the meristem of the inflorescence and observed the propagation of fractures. They showed that the distribution of compressive stress on the meristem of an inflorescence is consistent with the estimation based on the buckling model of Green [1,2]. This experiment does not directly demonstrate the effect of mechanical forces on the floral development, but the microsurgical manipulations could affect the internal stress distribution in the floral meristem.

### 2.2. Laser Ablation Experiment

Hamant et al. [6] published a milestone paper on the relationship between the developmental pattern on the floral meristem and mechanical forces. They confirmed that the orientation of the microtubule cytoskeleton dictates the developmental pattern on the floral shoot apex of *Arabidopsis thaliana*, which in turn is regulated by internal mechanical stress. They experimentally constrained the small floral meristem of *Arabidopsis thaliana* with two blades as Hernández and Green [12] did with the inflorescence meristem of the sunflower. The results showed that compression forces affect microtubule orientation on the meristem, which in turn may lead to the change in the developmental pattern of floral primordia.

**Figure 3:**
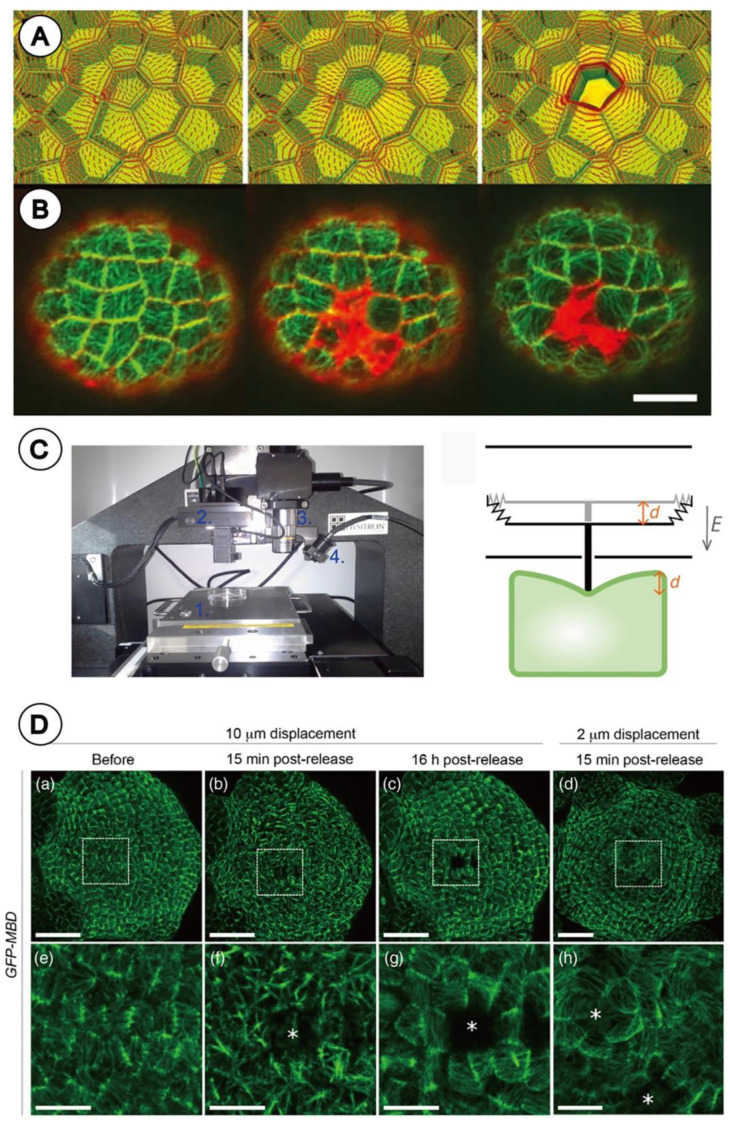
Laser ablation experiment (**A**,**B**) and nano-indentation experiment (**C**,**D**): (**A**) theoretical principal stress direction pattern (red lines) in epidermis cells of floral meristem before and after laser ablation. Color gives relative values of maximal stress (green, low; yellow, high). Left: a stress pattern in a group of cells before ablation. Middle: radial stress after drop of tensile stress in a ablated cell at center. Right: circumferential stress after weakening of top wall. (**B**) *GFP-MBD* expression (green) in epidermis cells of floral meristem in A*rabidopsis thaliana.* Left: before laser ablation. Middle: just after laser ablation. Right: 6 h 30 min after laser ablation. Ablated cells are shown in red (FM4-64 staining). (**C**) Nano-indentation. Left: indenter; (1) motorized stage; (2) tip and transducer; (3) × 10 objective lenses; (4) bright light. Right: drawing of transducer (bold black line) indenting a floral meristem (green object). When a voltage difference is applied between plates (thin black lines), an electrostatic field *E* is created, forcing middle plate to move over a distance *d.* (**D**) Microtubule response to compression by nano-indentation. Confocal images of *GFP-MBD* meristems projected in 2D. (**a**–**c**) Load value imposed during 6 h 30 min compression corresponds to an initial displacement of 10 µm; (**a**) before compression; (**b**) 15 min after release of compression; (**c**) 16 h after release of compression; (**d**) load value imposed during 6 h 30 min compression corresponds to an initial displacement of 2 µm; (**e**–**h**) close-ups of (**a**–**d**) (white rectangle). White asterisks point at ablation sites of GFP florescence. Scale bar in (**D**) = 30 µm (**a**–**d**), and 10 µm in (**e**–**h**) ((**A**,**B**) reproduced from Hamant et al., 2008 and from Louveaux et al., 2016 with permission) [6,14].

They also used a laser ablation approach to kill cells of the floral meristem to locally eliminate turgor pressure and weaken cell walls. The ablation induced changes in the microtubule orientation pattern on the floral meristem (Figure 3B) and this was consistent with the computed theoretical tensile stress direction pattern around the eliminated cells (Figure 3A). This approach demonstrated that not only the external mechanical forces, but also the change of the tensile stress pattern in the floral meristem by the laser ablation affect the developmental pattern in the meristem.

Louveaux et al. [14] also demonstrated that experimental perturbation of mechanical stress patterns by laser ablation affects the pattern of cell division plane orientation in the floral meristem of *Arabidopsis thaliana*. The selection of the plane of division usually involves a competition between alternative patterns whose geometries represent local minimum area (Besson–Dumais rule) [15]. However, Louveaux et al. [14] found that the Besson–Dumais rule worked well on those cells with isotropic tensile stress situated in the central part of the floral shoot apex, but not on the saddle-shaped cells with anisotropic tensile stress situated in the peripheral region of the apex. They induced the change in the internal tensile stress by killing cells of the floral meristem by laser ablation, and subsequently observed the meristem activity and identified which cells divided and the pattern of their division plane orientation. The results of the experiment demonstrate that the cells of which the direction of tensile stress changed did not follow the Besson–Dumais rule. This study demonstrated that mechanical forces affect the cell division.

### 2.3. Nano-Indentation Experiment

Beauzamy et al. [16] performed an experiment with mechanical forces on the floral meristem of *Arabidopsis thaliana* using the nano-indentation method (Figure 3C) and showed that the floral meristem behaves like a shell under about 1 Mpa of internal pressure. Louveaux et al. [17] developed this experimental methodology and successfully quantified the impact of a controlled and prolonged compression on microtubule behaviour in the *Arabidopsis* floral meristem, using microtubule fluorescent marker lines (Figure 3D).

### 2.4. Novel Experimental System Using Customized Microdevice

**Figure 4:**
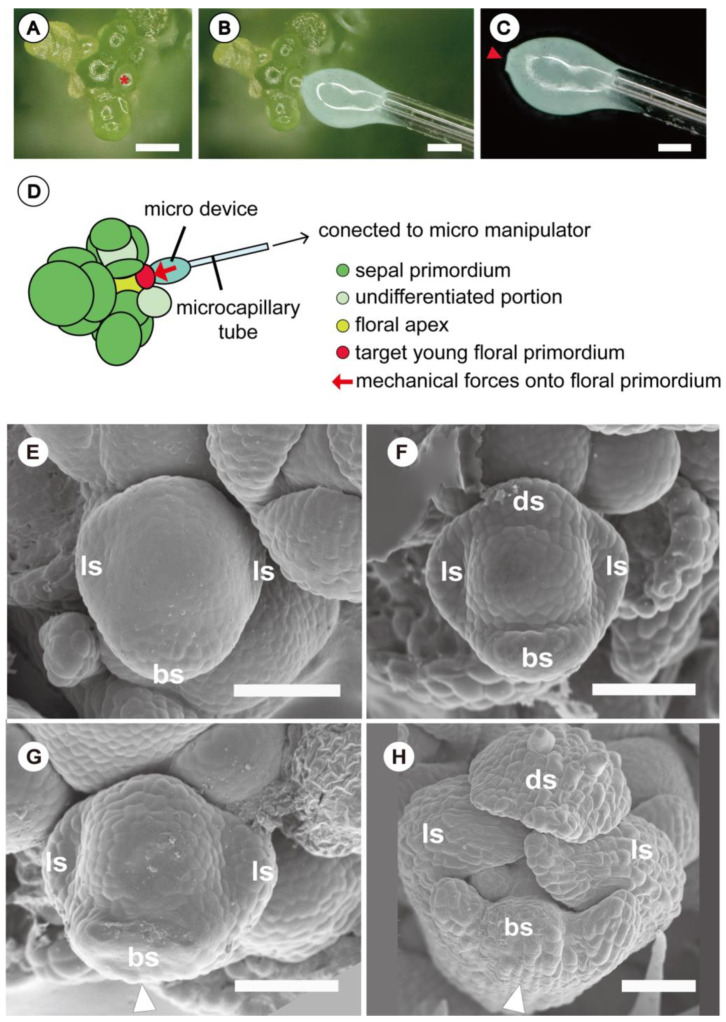
Novel experimental system with mechanical forces on a floral primordium of *Arabidopsis thaliana*: (**A**) dissected floral apex of *Arabidopsis thaliana* with a young primordium shown (*) prior to floral organ initiation. (**B**) Apex undergoing contact of unsolidified silicone paste on young floral primordium. After solidification of silicone paste, it becomes separated from floral primordium. (**C**) Separated silicone microdevice. Arrow indicates part that fits abaxial side of young floral primordium. (**D**) Schematic diagram of experimental system with effect of mechanical forces on floral primordium. Silicon microdevice (**B**,**C**) is pressed against abaxial side of floral primordium for more than 40 h to induce a mechanical force (several hundred µN). (**E**) Floral primordium at early stage without any external mechanical force. Abaxial and two lateral sepal primordia are initiated. (**F**) Floral primordium at a slightly later stage than that in (**D**). 3Abaxial and two lateral sepal primordia develop further, and an adaxial sepal primordium is initiated. (**G**) Floral primordium at an early stage after contact with microdevice for about 50 h. Note that the abaxial sepal primordium is deformed (white arrowhead) compared with that in (**E**). (**H**) Floral primordium after two days. Note that the tip of abaxial sepal primordium is three-lobed (white arrowhead) compared with that in Figure 4F. Abbreviations: bs, abaxial sepal primordium; ds, adaxial sepal primordium; ls, lateral sepal primordium. Scale bar in (**A**–**C**) = 100 µm, in (**E**–**H**) = 50 µm ((**A**–**H**) reproduced from Iwamoto, 2021 with permission) [19].

All these experiments presented above played with external mechanical forces or induced internal mechanical stress on the whole floral meristem, but none concentrated on a single floral primordium. Iwamoto et al. [18] developed a novel experimental system for the analysis of effect of mechanical forces on one floral primordium of *Arabidopsis thaliana*, aiming to induce morphological changes in the target flower. A micromanipulator with a microdevice shaped to fit the contour of the abaxial side of a young floral primordium was used to perform contact pressure (Figure 4A–D).

Morphological changes in the floral development of *Arabidopsis thaliana* were observed using this experimental system; for example, the tip of the abaxial sepal divided into three lobes when pressure was previously exerted on the corresponding floral meristem section (Figure 4E–H), demonstrating the shaping role that forces alone can have on a floral meristem. Iwamoto [19] integrated another experimental device for the measurement of minute mechanical forces in the context of this experimental system. This device, “Pollination Simulation”, was originally applied for the measurement of minute mechanical forces necessary for pollination [20], which successfully measured the mechanical forces on the floral meristem induced by the micro-device (in the order of several hundred µN). This novel experimental system successfully measured the artificial mechanical force on one floral primordium responsible for the morphological change of flowers. It is expected that further studies will elucidate the relationship between the strength of mechanical forces and the resulting pattern of floral morphological change.

## 3. Inferred Mechanical Forces Taking Place in the Bud

Floral organs develop within a bud enclosed by previously formed organs, leaves, and axes. The tight junction among structures and the observation of contact margins and associated shapes in the bud suggest the influence of forces on the flower configuration. Ronse De Craene [10] already treated the importance of pressures occurring in the inflorescence bud within his survey on the “physico-dynamic” aspects of flower development. The rationale here is that the sections of the floral bud that are under higher pressure are delayed in the inception and growth of an organ or prevented from initiating, while areas with less pressure would have a more rapid organ initiation and growth. We here give an overview of inferred pressures according to their spatial occurrence within the bud.

### 3.1. Bracts and Inflorescence Axis Pressing against the Flower Meristem

**Figure 5:**
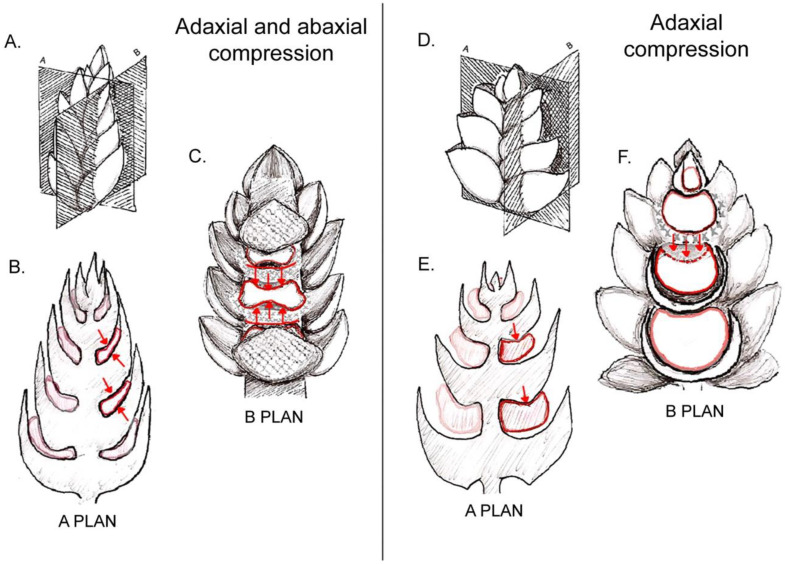
Examples of possible compression forces on floral primordia derived from subtending bracts and inflorescence axis: (**A**) scheme of an inflorescence bud and spatial orientation of sections shown in diagrams in (**B**,**C**). (**B**) Longitudinal radial section (corresponding to plan A shown in (**A**)) showing squeezed floral primordia as a consequence of a “sandwich” effect caused by compressing bracts. (**C**) Longitudinal tangential section (corresponding to plan B shown in (**A**)) revealing the elongated shape of floral primordia due to both ad- and abaxial pressure from bracts. (**D**) Scheme of an inflorescence bud and spatial orientation of sections of it shown in diagrams in (**E**,**F**). (**E**) Longitudinal radial section of inflorescence bud (corresponding to plan A shown in (**D**)) showing deformation on adaxial side of a floral primordium caused by its overlying bract. (**F**) Longitudinal section of inflorescence bud through plan B shown in (**D**). Note that the oppressed adaxial zone of the primordium is not capable of producing floral organs.

Lateral flowers are born from lateral meristems that develop in the axils of bracts. When the bract is of a rigid configuration, rapidly developing and tightly pressed against the axis, it reduces the available space for the axillary meristem and compresses the young developing flower against the axis. When the pressure of the bract is extensive, the floral primordium becomes squeezed and even flattened (Figure 5A–C). When the effect is weaker, the inhibition is restricted to one of the sides of the floral primordium, either abaxial (to the side of the bract) or adaxial (towards the inflorescence axis, Figure 5D–F).

#### 3.1.1. Compression and Flattening of the Flower Bud

**Figure 6:**
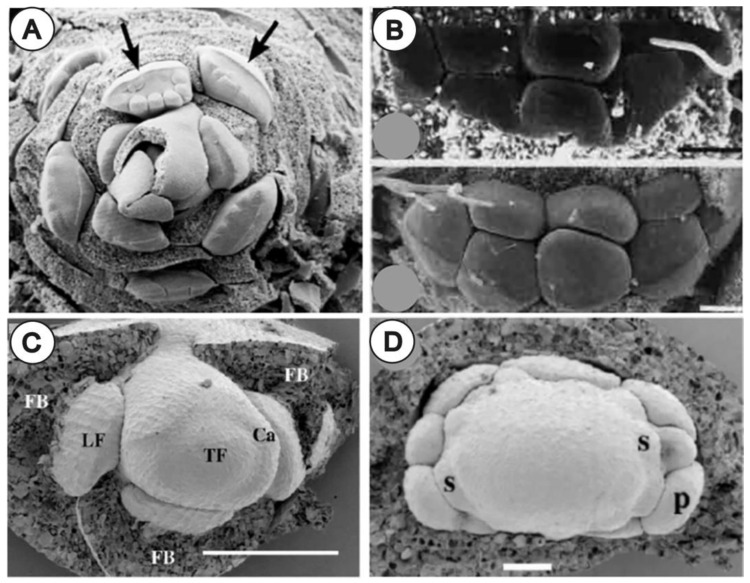
Examples of ellipsoidal compressed floral primordia and their mono- of disymmetric organ arrangement: (**A**) *Euptelea polyandra.* Note that scars are imprinted by bracts on the abaxial side (arrows) and earlier initiation of organs on adaxial side. (**B**) *Notobuxus natalensis*. Lateral staminate flower at two different stages of development. Note in (**A**,**B**) disymmetric arrangement of stamens along ellipsoidal floral primordium (**C**) *Drimys winteri*. Note: compressed flattened lateral flower primordia in contrast to terminal flower primordium, which is free of bract compressing forces. (**D**) Developing lateral flower primordium of *Drimys* showing its disymmetry (ellipsoidal flower base). In *Drymis*, lateral flowers have fewer organs than the radial terminal flower. Abbreviations: FB, floral bract; LF, lateral floral meristem; TF, terminal flower; Ca, calycine calyptra. Bar in (**B**,**C**) = 100 µm, (**D**) = 50 µm. ((**A**) reproduced from Endress, 2008; (**B**) from Von Balthazar and Endress, 2002; (**C**,**D**) from Doust, 2001 with permission) [7,23,24].

A tight pressure of the inflorescence bracts can mold monosymmetric lateral buds (Figure 5B,C), as seen in the flowers of *Euptelea* (Eupteleaceae, Figure 6A) [7,21,22], *Notobuxus* (Buxaceae, Figure 6B) [23] or *Drymis* (Winteraceae, Figure 6C,D) [24]. Similarly, the tetragonal lateral flowers that occur in Bataceae and Salvadoraceae seem to be a direct consequence of the compression of their floral meristem between bract and inflorescence axis [25,26].

In inflorescences that possess terminal flowers, this phenomenon is especially manifested since terminal flowers are not squeezed by a subtending bract as they develop directly from the transformation of the inflorescence meristem [27]. For example, terminal and lateral flowers of *Styloceras* and *Notobuxus* (Buxaceae) differ in shape and sex distribution [23]. Lateral flowers in *Drymis* (Figure 6C,D) [24] present lower floral organ number and an altered phyllotaxis. There is also a different merism in lateral versus terminal flowers of *Trochodendron, Ruta, Adoxa,* among others [10,22].

In Poales, there is also evidence of organ variation due to tight pressures of subtending bracts. Here, floral reductions are documented in correspondence with a shift to wind pollination correlated with tight inflorescences and a carpel reduction and loss. These take place mainly in a median direction, suggesting a flattening of the floral primordium by the subtending bracts (e.g., Restionaceae [28]; Poaceae [29]). Also, Reynders et al. [30] demonstrated that dimerous gynoecia in Cyperaceae are derived from trimerous gynoecia that are dorsiventrally flattened; thus, the pressure of flowers between bract and axis leads to the loss of the adaxial stamens and median carpel. They also referred to laterally flattened gynoecia, but here the compression leads probably to the development of styles in novel positions, where there is more space for them to develop.

#### 3.1.2. Adaxial or Abaxial Inhibition

**Figure 7:**
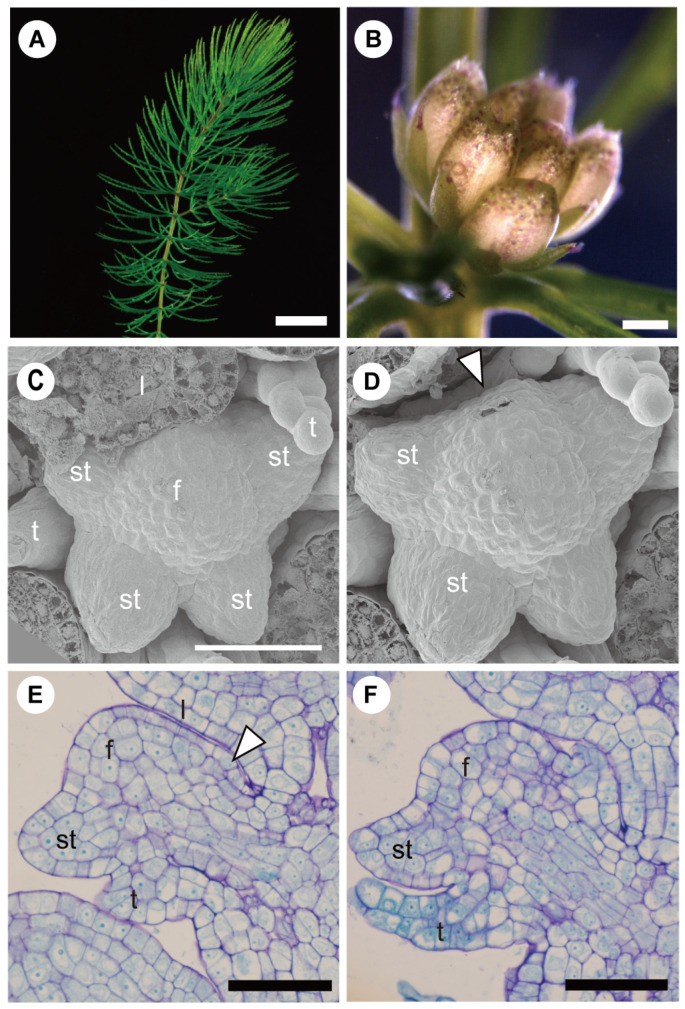
Adaxial developmental inhibition in staminate flower of *Ceratophyllum demersum*: (**A**) shoot of *Ceratophyllum demersum* in aquatic environment. (**B**) Staminate flower consisting of numerous stamens and surrounding tepal/bracts. (**C**) Staminate floral primordium at an early stage. Abaxial and lateral stamen primordia are initiated. Adaxial side of primordium is covered (contacted) with leaf primordium. (**D**) Same floral primordium in (**C**). Leaf primordium at adaxial side of floral primordium was removed. No stamen primordium is initiated at the adaxial side (white arrowhead). (**E**) Stamen and tepal/bract primordium initiated on the abaxial side and just bulge found on the adaxial side (white arrowhead) in staminate floral primordium at an early stage. Note that an upper leaf primordium tightly contacts with the adaxial side of floral primordium. (**F**) Staminate floral primordium at a slightly later stage than that in (**E**). Tepal/bract primordium is initiated on the adaxial side, but no stamen primordium is initiated on same side. Abbreviations: t, tepal or bract primordium; f, floral apex; l, leaf primordium; st, stamen primordium. Scale bar in (**A**) = 5 mm, in (**B**) = 500 µm, in (**C**–**F**) = 50 µm. ((**A**,**B**,**E**,**F**) reproduced from Iwamoto et al., 2015; (**C**,**D**) from Iwamoto et al., 2003) [31,32].

The effect of pressure of the bract can be expressed on either side of the floral primordium along a median gradient, favoring a polarity in flower sequence initiation and development, and eventually also organ loss.* Ceratophyllum* presents a good example where the effect of the pressure is more pronounced on the adaxial side of the flower primordium (Figure 7) [31,32]. Here, the bract subtending a staminate flower overtopping the floral bud presses against the adaxial side and stamens may fail to develop as organs can only initiate unidirectionally from the abaxial to the adaxial side (Figure 7D–F).

In analogy, in many monosymmetric flowers the organ initiation sequence tends to be unidirectional, mostly running from the abaxial to the adaxial side (many Leguminosae, Lamiales), and this is probably related to the internal pressures becoming stronger towards the inflorescence axis (Figure 5D–F). For the Leguminosae this could be related to the unusual shift of the odd member of petals to the median abaxial position [33], likely changing the direction of forces in morphogenesis. Monosymmetry is genetically controlled by a gradient in the expression of *CYC*-like genes [34], which is triggered by gravitational forces and the heterogeneous pressure of the bract on the floral meristem, as pointed out by Ronse De Craene [10].

In some cases when bracts are more reduced in size and the packing within the inflorescence bud seems to be looser, the pressurizing effect of the subtending bract appears to be restricted to the abaxial side of the floral meristem. This is in turn related with an inversion of the floral initiation sequence from the adaxial to the abaxial side (e.g., *Cyphia* [35], *Lobelia,* see Figure 11F in [27]). This might have structural consequences in the mature flower since under this inverted organ initiation sequence, the median sepal initiates in abaxial position, and consequently, the median petal in an adaxial position, giving rise to an inversed floral display, e.g., as in the case of *Cyphia* [35].

Also, a more extensive adaxial development is observed in the staminate inflorescences of *Batis*, where the calyx develops as a bilobed saccate structure with the adaxial lobe more strongly developed as a scale where pressure of the bract is less strong [25].

However, it is not possible to generalize patterns of floral initiation sequences as there is variability even among species within the same family, ranging from adaxial to abaxial or even bidirectional [36,37]. This should be an incentive to perform critical observations in species with inversed initiation sequence to investigate a potential correlation between the bud configuration and influence of the subtending bract. Other factors besides the pressure of bracts necessarily influence the floral initiation and the eventual development of monosymmetric flowers. The influence of the bracts on flower development may also fluctuate during the development leading to different shapes of mature flowers.

#### 3.1.3. Further Phenomena of Compression between Bracts and Axes

Foliar organs other than the flower subtending bracts can also exert contact pressure on the developing flower primordium within the inflorescence. Naghiloo et al. [38] illustrated how the pressure of subtending leaves of the compound inflorescences of *Astragalus* (Leguminosae) influences the development of flowers. Here, the flowers have their first sepals always initiated in areas that show the least pressure. Similarly, the initiation sequence within umbels of Loteae is dependent on the asymmetric shape of the inflorescence primordium, which is manifestly shaped by the tightly compressed leaves surrounding the inflorescence [39,40].

### 3.2. Involucra in Flowers, Floral units, and Inflorescences

**Figure 8:**
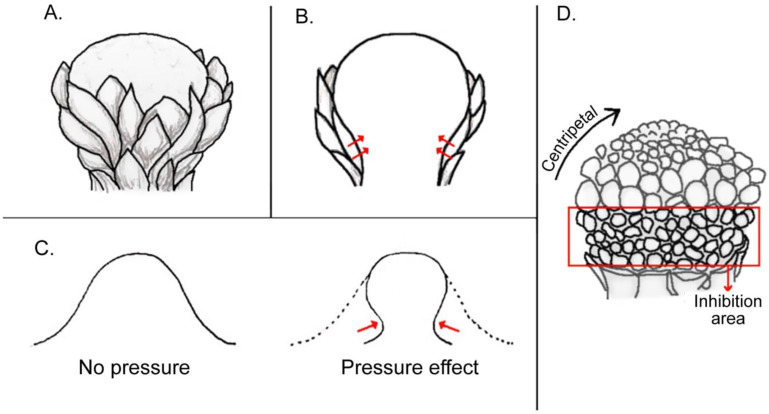
Illustration of compression forces of an involucrum on a central meristem: (**A**) figure of an involucrum formed by several bracts surrounding a central reproductive meristem. (**B**) Longitudinal section of (**A**), showing pressure exerted by involucrum at the base of the meristem (red arrows). (**C**) Diagram of a typical FUM, i.e., a head meristem, showing centripetal progression of florets in the upper part and delayed floret growth at its base in area of inhibition covered by involucrum. (**D**) Silhouette of a terminal meristem without (left) and with an involucrum (right). Red arrows denote deformation of the meristem by an involucrum.

**Figure 9:**
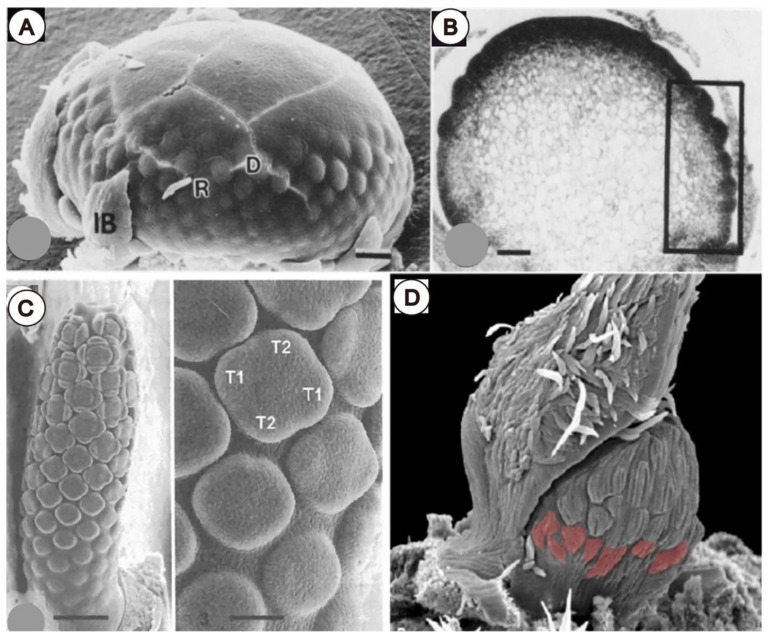
Examples of inhibition of basal organs by pressure exerted by involucral bracts: (**A**) delayed development of florets at base of capitulum of *Erigeron philadelphicus* where bracts were removed, as seen under scanning electron microscopy (SEM). (**B**) Same as (**A**) as a longitudinal histological section, highlighting in box compression zone of involucral bract that shows a basipetal inhibition of floret primordia. (**C**) Basal floral primordium inhibition in spadix of *Anaphillopsis americana*. (**D**) FUM of *Davidia involucrata* showing in red delayed floral primordia at its base. Abbreviations: IB, involucral bract; R, ray flower primordia; D, disk flower primordia; T1, lateral tepal primordium; T2, median tepal primordium. Scale bar in (**A**,**B**) = 50 µm, in (**C**) (left) = 300 µm, in (**C**) (right) = 75 µm. ((**A**,**B**) reproduced from Harris et al., 2005 with permission; (**C**) from Barabé and Lacroix, 2008; (**D**) from Jerominek et al., 2013) [47,50,53].

Specialized foliar organs such as an involucrum and spathe may surround flower-like inflorescences, which are known as ‘floral units’ [41] (such as heads of Asteraceae, umbels of Apiaceae, spadices of Araceae, etc.) and play an important role in the early development of flowers (Figure 8). While these structures are linked to the protection of the developing reproductive organs and attracting pollinators (e.g., [42,43,44]), in manycases they may also play a decisive developmental role by exerting a compressing force on the enclosed developing young flowers (Figure 8). As the major pressure of involucra and spathes occurs at the base of the floral unit close to the attachment point (Figure 9A,B,D), the consequence is a retardation of the initiation and/or growth of the basal-most flowers. This was documented in the development of the spadices of Araceae (Figure 9C) [45,46,47] and *Gunnera* [48], as well as in the heads (capitula) of Asteraceae (Figure 9A) [49,50,51,52] and of *Davidia* (Figure 9D) [53]. Interestingly, the retardation and inhibition of basal florets in heads of Asteraceae coincides with their identity as ray florets (Figure 8 and Figure 9) [50]. In this regard, it could be hypothesized that the pressure of involucral bracts on the basal florets of the asteracean head is a key ontogenetic trigger for their configuration as ray florets (Figure 8), probably even triggering the corresponding genetic cascade that leads to their differentiation. This pressure may also affect sex differentiation, since ray florets are sterile in some taxa (e.g., *Helianthus annuus* L. [54]). Conversely, it could be also inferred that capitula lacking ray florets would develop from buds lacking a tight involucrum, and consequently, a lack of pressure in the basal-most part of the capitulum primordium.

Uncommon cases of inflorescences or floral units commonly known to bloom in basipetal (centrifugal) direction, such as *Dipsacus* (Caprifoliaceae), *Gunnera* [48], *Orchys simia* (Orchidaceae), *Petasites*, *Liatris*, *Echinops* (Asteraceae), and some grasses as *Urochloa* [55], could be also related to retardation of flower primordia located towards the base. Despite the fact that ontogenetic studies are not available for all these species, tightly enclosing bracts surrounding the inflorescence bud is commonly seen (unpublished observation).

In some flowers, involucra can be found as foliar structures below the calyx. The flowers of the Portulacineae clade of Caryophyllales (except Cactaceae) are characterized by the presence of two large median involucral bracts. The compression of flowers between involucral bracts is very strong in Montiaceae, especially in *Lewisia*, creating a stronger pressure on the median axis of the flower, giving rise to an ellipsoid flower primordium, and leading to an increase in petaloids and stamens. Lateral stamens that arise on the transversal axis, where the compression force is expected to be less strong, grow more strongly than the ones in a median position [56]. The pressure caused by these bracts in early stages of development causes deep disruptive changes in the phyllotaxis. In *Lewisia*, these changes lead to a higher number of organs (stamens and petaloids). In *Claytonia*, on the other hand, the compression affects the whole meristem, leading to constraints that result in the annulment of petaloids, which start growing at a much later stage from filament tissue [57].

**Figure 10:**
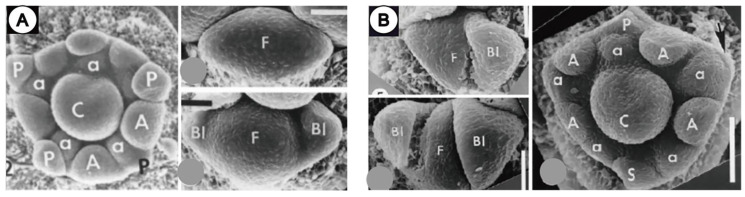
Inferred effect of bracteole compression on flower primordium in Fabaceae. (**A**) Development of *Schotia brachypetala* showing “circular” flower type with bracteoles that are relatively small and of loose contact with inner floral meristem, producing a mature flower with 5 petals and 10 stamens. (**B**) Development of *Aphanocalyx djumaensis* of “omega” type, with bracteoles relatively larger in size and in tight contact with floral meristem that develops usually only 1 abaxial sepal and 1 adaxial petal and 9 stamens (petal rudiment shown in arrow). Compression exerted by bracteoles is noticed by pressure scars and squeezed form of the floral meristem in D, also evidencing lack of petal primordia in lateral abaxial positions of typical ‘‘keel’’ petals of Fabaceae. Abbreviation: F, floral meristem; Bl, bracteole; P, petal primordium; A, external anthers; a, internal anthers; S, sepal primordium; C, carpel. Scale bar in (**A**,**B**) = 50 µm. ((**A**) reproduced from Tucker, 2001; (**B**) from Tucker, 2000 with permission) [58,59].

Bracteoles, i.e., the one or two small leaves carried on the petiole of a flower in lateral position, could also adopt an involucral behavior. Bracteoles usually do not compress the flower massively, but in certain cases they can also be responsible for space constraints leading to loss of organs in the flower [10]. Clear examples for this are presented by several studies of the floral development of Caesalpinioid legumes by Tucker [58,59,60], who demonstrated the existence of two kinds of flower developmental patterns linked to bracteole development. The “circular type” pattern involves the floral bud with two small lateral bracteoles present and five sepals that arise in a helical sequence. In contrast, the “omega-type” pattern is characterized by the existence of large-fused bracteoles that tightly enclose the floral meristem. Here, only one sepal and one petal fully develop with altered organ initiation sequence (Figure 10) [58]. Furthermore, the tightly pressed bracteoles—that adopt a spathe form at their base—are to some extent responsible for the organ suppression (Figure 10).

Bracteoles exert an influence on the direction of development of the calyx, which may in their turn influence the position and sequence of initiation of the petals [61]. Based on the floral development of *Melanophylla*, the authors questioned whether mechanical forces are the only factors influencing the direction of contortion of the petals, rather suspecting a prepatterning influence of the sepals. However, their observations relate to the shape of the petals, not to the sequence of initiation.

### 3.3. Within-Flower Organ Interaction

#### 3.3.1. Influence on Organ Position

Early initiated organs have a decisive influence on the initiation of neighboring internal organs, affecting their position, size, symmetry, and developmental rate [10]. There are many examples of such influence, and it seems that the organs that undergo pressure are mostly sepals, petals, and stamens. The pistil, being the most central organ in the flower, and usually the largest, tends to greatly influence the arrangement and fate of neighboring organs. Even though the pistil is the last organ to be formed, it seems that its inception area already influences the position of the remaining organs in the flower through a basipetal prepatterning process [62,63]. The fate of the pistil, in its turn, is influenced by the size of the floral apex, affecting the number of carpels that can be initiated [10,64,65].

The controversy around obdiplostemony was discussed by Ronse De Craene and Bull–Hereñu [66] as a matter of spatial constraints. Secondary obdiplostemony represents a typical condition where the mutual position of the two stamen whorls shifts during development. The pressure of the fast-developing calyx will push antesepalous stamens inwards and induce the antepetalous stamens to move outwards (in relation to a delayed petal development and carpel pressure when isomerous). In isomerous flowers of Caryophyllaceae, the position of carpels fluctuates between antepetalous or antesepalous, depending on the position of the upper stamens, which are in turn influenced by the extent of development of the calyx [67]. When sepal development is delayed and more or less simultaneous, they exercise less pressure on the antesepalous stamen whorl and carpels are inserted in antepetalous position. With a strong pressure of the sepals due to a disjunct spiral 2/5 development, antesepalous stamens are shifted more centrally and carpels become inserted in antepetalous position.

These shifts demonstrate that floral organ initiation and development remains highly flexible and depend on internal forces regulating the spatial arrangement of the organs.

#### 3.3.2. Shifts in Developmental Sequence and Organ Loss

Expansion and fusion of sepal primordia often results in petal loss. Interestingly, the *Arabidopsis* mutant *petal loss* (*ptl*) that lacks petals in its flowers, is also characterized by overgrown sepals (Lampugnani 2012). The target gene *PTL* codes for a transcription factor expressed in sepal boundaries, i.e., it inhibits the tissue proliferation between sepal primordia, keeping them as isolated units [68]. In the wild-type flower meristem, petal primordia arise in alternation with the sepals, i.e., adopting a position in the gaps between sepals. In the absence of *PTL*, however, no boundary between sepals is maintained and the gap between them is filled with sepal tissue [68]. Under the rationale of this review, it could be hypothesized that in the *ptl* flower, the incremental growth in the sepal whorl could mechanically obstruct the initiation of petal primordia on the floral meristem. This point of view could explain the absence of petals in the *ptl* mutant even if the target gene is acting in a different whorl. However, other mechanisms may be at play leading to petal loss, such as the homeotic transformation of petals in stamens in the *spe* mutant of *Capsella bursa-pastoris* [69] or the apetalous *lel* mutant, where loss of petals is the result of a progressive abortion [70].

A trimerous gynoecium often generates the loss of organs lying in the radius of the carpels, linked to the outwards spatial constraint that the carpels create. Stamen reduction and loss is often predictable and depends on the external pressure of the sepals and the internal spatial occupation of the ovary. Studies in Caryophyllales showed that stamen loss is inversely related to the sequence of development of sepals and depends on the number of carpels being produced (see [71,72,73]). For example, with three carpels, inner stamens will be opposite the three inner sepals, while with two carpels, two inner stamens are always opposite sepals four and five (with a 2/5 arrangement of the sepals). A similar reduction is observed in *Frankenia* where four antepetalous stamens are lost and the remaining stamens are rearranged as two trimerous whorls [33].

There are several other examples demonstrating the pincer effect of carpels and sepals. In *Tripetaleia* Ericaceae), stamens and petals are trimerous and this is induced by the ovary [74]. In Cistaceae, the genus *Lechea* has a trimerous androecium and petals squeezed between three larger sepals and three carpels [75]. In some cases, the trimerous ovary can induce a reduction in stamen numbers from five to three by the pairwise fusion of four stamens lying in the radii of the carpels. The resulting stamens alternate with the carpels, and this was observed in *Hypericum* (with the development of stamen fascicles) [76] and *Mollugo* [77].

The octomerous androecium of Sapindaceae is strictly correlated with a trimerous gynoecium, where stamens become accommodated in the limited space of the floral bud [78]. The generalized loss of two stamens in the family is linked with the pressure of carpel sectors in a confined space, and this pattern became stabilized in evolution. Other examples are presented in [10,79].

The pressure of perianth organs on the androecium influences the initiation sequence of stamens, their position, and possible reduction. In diplostemonous androecia, stamen initiation usually proceeds in a centripetal direction. However, pressures in early stages of development often result in the centrifugal inception of stamens as is visible in some obdiplostemonous taxa [66]. Polyandry can be influenced by mechanical pressures caused by the perianth and a precociously developing gynoecium. Several polyandric species (especially in Cornales and Ericales) show a limited centrifugal or lateral proliferation of stamens from initial stamens developing on a ring primordium. The space limitation between a more strongly developed gynoecium and perianth squeezes additional stamens in loops or girdles around stamen forerunners (e.g., *Actinidia* in Actinidiaceae [80]; *Carpenteria* in Hydrangeaceae [81], *Napoleonaea* in Lecythidaceae [82]). When extension of the receptacle is less limited and sufficient space is available, a secondary stamen increase is less constrained and runs either centripetally or centrifugally [83].

In *Phytolacca,* the carpel whorl is increased to 8 or up to 10 carpels by expansion of the floral apex. This has a direct influence on the number of upper stamens that become accommodated in the spaces between the carpels, fluctuating between 5 and 10 [84].

Similar to the pressure exercised by bracts, a faster development of the abaxial sepals can lead to early monosymmetry and a retardation of the adaxial side of the flower, including sepals in some species of *Cleome* [85,86], while species with subequal sepal development retain the dissymmetry common to core Brassicales. However, at maturity all species share a comparable monosymmetry [86].

Contortion of the perianth is also a factor causing mechanical pressure. In Cistaceae, the calyx is often differentiated in two smaller outer and three larger inner sepals. The petals are contorted in opposite direction to that of the larger sepals, resulting in a shifted position [75]. In several Malvaceae, contortion of the petals strongly influences the development and position of the stamens, which may appear shifted in position [87,88].

#### 3.3.3. Changes of Shape Induced by Outer Organs

The pressure exerted by external organs mold the shape of the organs they cover. This closely relates to the concept of “imprinted shape” by Endress [7,89,90,91], in which floral parts are shaped by adjacent organs during development leaving pressure marks recognizable on the tissue [7].

Inner organs in bud often show the pressure marks of the perianth. For example, the pressure of the contorted petals is visible on stamens and stigma in *Conostegia* [92]. and the pressure of the perianth may affect the development of an androecial member as part of the labellum in Zingiberaceae [93]. The surface of the ovary can also be imprinted by stamens. For example, ovaries of *Ophiocaryon* (Sabiaceae) have a rough upper surface and smooth lower surface, of which the latter could be formed by pressure from surrounding stamens and staminodes [94]. The young stigma of *Asclepias* is initially two-lobed, reflecting the bicarpellate nature of Apocynaceae, but pressure of neighboring pentamerous organs causes the stigma to become pentangular [7,95].

Pressure can also inhibit the initiation of carpels as in *Psilopeganum* (Rutaceae), where only two carpels are initiated, while the floral anatomy shows evidence of four carpels, which is a more common carpel merism in Rutaceae, together with pentamery [96]. A similar reduction was reported for the gynoecium of *Pelliciera* (Tetrameristaceae), having five styles but only two locules [97]. The monomerous gynoecium of Lauraceae is molded by the preceding trimerous whorls (see citations in [90]).

The shape of sepals may dramatically change during development. This is particularly clear for the initiation of a valvate calyx. In cases where sepals initiate as broad triangular organs, the curvature of the margins shape the bud (e.g., [7]) but also put greater pressure on sectors lying in the radius of the sepals. As a consequence, the floral bud may become star-shaped with a stronger development of the alternisepalous sectors (e.g., *Elaeocarpus*: Bull–Hereñu and Ronse De Craene unpublished data). Flower buds with early differentiated valvate sepals are generally strongly angular (e.g., Primulaceae, Lecythidaceae, Rosaceae). If the pressure of the valvate calyx is not too strong, this can lead to a greater elaboration of petals, as the development of appendages is not much obstructed [7].

Valvate sepals may also develop in later stages of development by equalization of sepals arising sequentially with a quincuncial aestivation (e.g., Leguminosae, Myrtaceae) [98,99,100]. In this case, their impact on the floral bud and the position of inner organs will be less dramatic.

The molding pressure induced by external organs may act at different stages of development. In early stages of development, the outer median calyx lobes will create disymmetry of the flower as in Papaveraceae, Begoniaceae, Brassicaceae (similar to involucral bracts). Additionally, pressures from the central meristem that give rise to the ovary have an increased influence during the development of the flower. The disymmetry in the flowers of species within Begoniaceae, Papaveraceae [101], *Theligonum* in Rubiaceae [102], and Brassicaceae [85] can also be traced back to an elliptical form of their floral primordium. Sepals could be acting in a similar way to the involucre of Portulacinae (see Section 2.2), molding the elliptical form of the floral bud.

**Figure 11:**
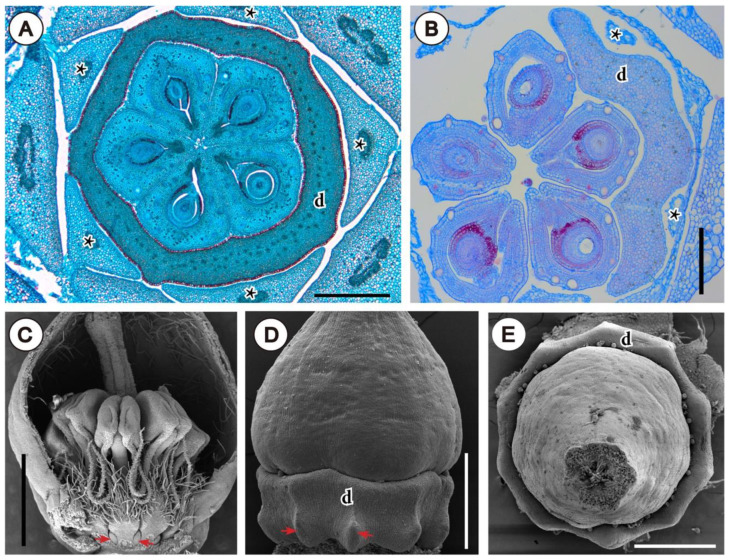
Inferred effect of spatial interaction of organs within young flower. (**A**,**B**) Photomicrographs of microtome transections of floral buds of Rutaceae, close to the floral base. (**A**) Detail of *Conchocarpus macrocarpus* (Engl.) Kallunki & Pirani, note polygonal shape of nectary and five protuberances between filaments. (**B**) Detail of *Ertela bahiensis* (Engl.) Kuntze, at mid-level of ovary; note outline of unilateral disc. (**C**–**E**) SEM micrographs of flowers of *Arctostaphylos pungens* (Ericaceae). (**C**) Bud longitudinally opened; arrows indicate basal protuberant part of nectary formed between filaments. (**D**) Lateral view of ovary and nectary (stamens removed), with arrows indicating same region as in (**C**). (**E**) Same as in (**D**), frontal view, showing decagonal outline of nectary disc. Abbreviations: asterisk, filaments; d, nectary disc. Scale bar in (**A**,**D**,**E**) = 500 μm, in (**B**) = 200 μm, in (**C**) = 1 mm.

Furthermore, nectary shape can be molded by the pressure exerted by previously formed organs during development. This can be observed in buds of Rutaceae, where space availability is restricted at the moment of nectary growth, which occurs usually late in floral development [103]. Such “imprint” in the outline of the annular intrastaminal nectaries can be observed in some Galipeinae of Rutaceae, mostly due to the pressing of filaments and petals in its outer side, and to carpels in its inner side (Figure 11A,B; See also Figures 1D and 3G in [104] and Figures 6I, 16G1 and 16H1 in [105]). Similarly, in *Arctostaphylos pungens* Kunth (Ericaceae), this is also clear in the basal protuberances of the nectary, formed only between stamen filaments, where more space is available (Figure 11C,D). Also, the decagonal shape of the nectary (from above, Figure 11E), is created due to the pressing of the 10 filaments (El Ottra and Nogueira, unpublished data).

## 4. Non-Intrusive Visualization of Flower Development and the Outcome of Mechanical Forces

As shown above, there are many structural interactions that result in mechanical forces influencing the development of the entire flower or a particular whorl or organ. However, one major drawback in studying the spatial relations between organs is the necessity to dissect or section the flower to usually study only the outcome of mechanical forces. Direct observations of development are naturally limited to the external parts of the developing flower (usually bracts, bracteoles or perianth organs). The often destructive and indirect way of visualization still allows for an interpretation. However, the methods are prone to introduce misinterpretations or result in overlooking important clues given by spatial relations between organs.

An opportunity to study organ interactions as a proxy of mechanical forces during development is to study floral organs during development in their natural configuration. Modern 3D-imaging techniques have great potential for the visualization of floral structures in situ or in vivo and might revolutionize our understanding of mechanical forces in flower development.

Techniques relatively recently introduced to plant science and now commonly applied are X-ray micro-computed tomography (µCT) [106] and X-ray microscopy (XRM) [107,108]. These X-ray techniques allow for the visualization of intact flowers and floral buds in 3D. These include a wide range of materials, ranging from heavy metal stained critical-point dried [109] to freshly detached (i.e., fresh [106]) flowers, and even flowers and inflorescences still attached to the living and growing plant can be studied (e.g., [110]). Additionally, fossil flowers can be studied morphologically and anatomically to some extent without destroying the fossils in the process (e.g., [111,112]). However, the techniques require the samples to be stable over a relatively long period—a couple of minutes to several hours, depending on sample size, required resolution, and type of scanner [108,109]. This usually requires the flowers to be chemically fixed (e.g., in FAA or ethanol), and subsequently dehydrated and CP-dried or embedded in a medium such as low-melting point agarose [108,109]. In the most common case of fixed samples, observations of development are indirect; i.e., development cannot be directly observed, and several developmental stages of different flowers need to be scanned. Even if living flowers are used, the long duration of high-intensity X-ray radiation is likely to introduce changes in development and artifacts are introduced in the scan results due to movements of the sample [110]. Nevertheless, one can assume that observed flowers remain similar or close to their natural configuration while being scanned [107,108]. The data then can be analyzed without using a destructive approach. Sectioning and dissection (segmentation) can be performed with limited loss of information using the dataset and specialized software.

**Figure 12:**
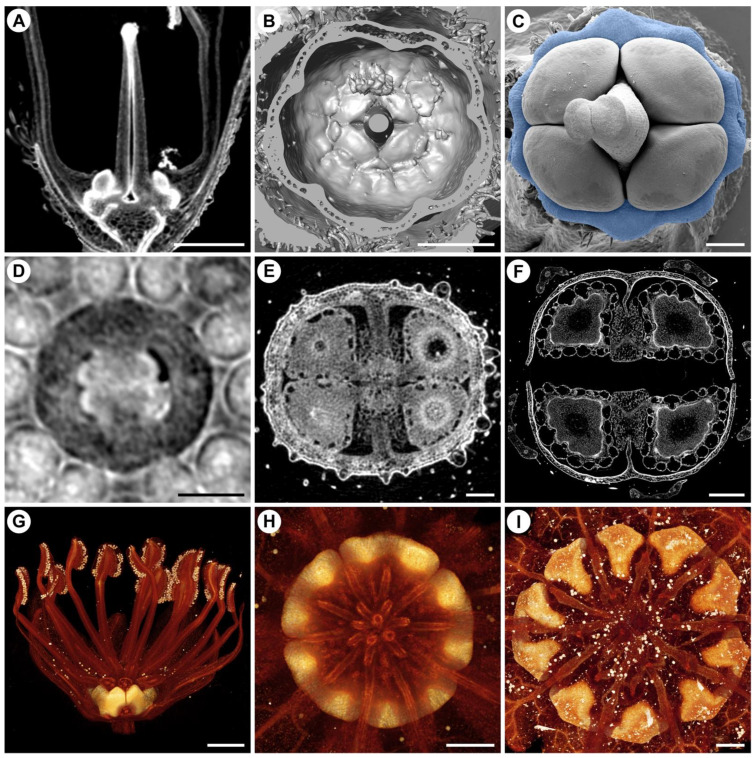
Flower structure and development visualized using µCT. (**A**–**C**) *Myosotis sylvatica*. (**A**) Virtual longitudinal section through an anthetic flower showing integration between nectary disc, mericarpids, and basal scales at base of corolla tube. (**B**) 3D-surface visualization of same data as A showing 10 basal scales enclosing ovary. (**C**) SEM image of gynoecium with lobed nectary disc highlighted in blue. (**D**–**F**) *Phacelia malvifolia*. (**D**) Virtual cross-section through young gynoecium at stage of ovule initiation. (**E**) Virtual cross-section through ovary at anthesis. (**F**) Virtual cross-section through mature fruit. (**G**,**H**) *Croton chilensis*. (**G**) 3D-volume rendering of virtual tangential-section through a staminate flower showing position of nectary glands between stamens and petals. (**H**) 3D-volume rendering of a staminate flower with upper part of stamens virtually removed. (**I**) *C. alabamensis*. Three-dimensional-volume rendering of a staminate flower showing presence of 10 nectary glands. Scale bars: (**G**) = 1 mm, (**A**,**B**,**F**,**H**,**I**) = 500 µm, (**C**–**E**) = 100 µm. (**A**–**C**) from Jeiter et al. (2020; published by Oxford University Press in Botanical Journal of Linnean Society, Volume 193, Issue 1, https://doi.org/10.1093/botlinnean/boz112, accessed on 1 November 2021), copyright Oxford University Press. (**D**–**F**), copyright Maria–Anna Vasile 2021, licensed under CC NC-BY (https://creativecommons.org/licenses/by-nc/4.0/, accessed on 1 November 2021), adapted from Vasile et al. (2021; published by Wiley in American Journal of Botany at https://doi.org/10.1002/ajb2.1691, accessed on 1 November 2021). [113,116] (**G**–**I**) modified from unpublished data of P. Thaowetsuwan.

An example for the application of µCT to study the outcome of mechanical forces is the study of faucal and basal scales in Boraginaceae [113]. Faucal scales result from invaginations of the stamen–corolla tube, while basal scales are protuberances of the same tube. Both types of scales are initiated in late flower development. Faucal scales are inserted at the level of the anthers. They occupy the space between the anthers and only develop into their final shape, size, and position in late development, when the floral bud enlarges and elongates. Differently, basal scales are initiated between the base of the anthers and the base of the ovary, close to the nectary disc. At initiation, the basal scales occupy the entire space between anthers and nectary disc. At anthesis, the fully developed basal scales are tightly pressed against the ovary, usually covering the entire nectary disc and reaching between the typically four mericarps. The tight fit between basal scales and ovary influences the shape of the nectary disc, which becomes lobed, and the shape of the basal scales, which have to fit in between the mericarps. Since there are usually four mericarps and 10 basal scales, the shape and size of the basal scales varies depending on their relative position to the mericarps [113]. The spatial relation between scales and the floral organs is best resolved with µCT (Figure 12A–C).

A similar relation between modifications of the stamen–corolla tube and nectary disc can be observed in Hydrophyllaceae (Boraginales) [114]. Here, the nectary disc often forms five lobes or even separate glands that are tightly enclosed by paired corolla scales. Both the development of the glands and of the corolla scales is correlated in time and space resulting in their tight fit and functional integration in the anthetic flower. A similar phenomenon was observed in *Codon* (Codonaceae, Boraginales) [115].

In another study on Hydrophyllaceae, the relation between seed shape and ovary shape became apparent [116]. In this study, the development of the internal ovary architecture was studied using µCT. The study of ovule and seed development showed that seed shape, especially in four-seeded fruits was often triangular in cross-section while developing from initially round ovules ([116]; Figure 12D–F). The shape of the mature seeds is determined by the restricted space inside the ovary. However, this applies only to a few species. In other, also four-seeded species, the developing seeds maintain their round shape, indicating that other factors than mechanical forces play a role in seed shape formation.

Furthermore, µCT also reveals the diversity of floral nectaries in the megadiverse genus *Croton* (Euphorbiaceae, Malpighiales), especially in staminate flowers. Nectaries in staminate flowers in most *Croton* species consist of five separate glands alternating with the petals and the outermost antepetalous whorl of stamens (Figure 12G,H). However, in the genetically isolated *C. alabamensis*, the nectary consists of 10 glands that are sometimes fused forming five horse-shoe shaped glands clasping the base of the outermost alternipetalous stamens (Figure 12I) [117]. Previous ontogenetic studies found that the nectary is the last structure to develop in the flowers of *Croton* [117,118]. Thus, the presence of alternipetalous nectary glands in *Croton* is likely due to the availability of space in between the previously developed petals and stamens at the onset of their development. Moreover, staminate flowers of *C. alabamensis* are cup-shaped due to the presence of a hypanthium, which is unique among all *Croton* species [117]. The formation of a hypanthium provides more space between the petals and outermost stamen whorl than in *Croton* flowers lacking a hypanthium. Therefore, the nectaries in staminate flowers of *C. alabamensis* are possibly in a shifted position and increased in number, as there is no spatial constraint hindering their development.

µCT is a powerful and fast tool to study and visualize flower development and the outcome of mechanical forces, but as shown in the studies above, it is most powerful if combined with other traditional techniques, such as SEM and light microscopy.

Another promising technique that only recently gained more attention for basic research is magnetic resonance imaging (MRI or NMR: nuclear magnetic resonance). MRI is a complex imaging technique that uses the magnetic resonance of specific atoms for visualization. A short explanation on the principles of the technique and a collection of helpful literature can be found in the review by Borisjuk et al. [119]. MRI was used in plant science since the early 1990s mainly to monitor bulb and fruit development in vivo and in situ (e.g., [120,121]). Recent developments of MRI for applications in plant science increased the potential voxel resolution close to 1 µm [119,122]. The benefit of MRI over µCT is that the samples do not have to be as stable and that contrasting with heavy metals to improve resolution or to differentiate between tissue and background is not necessary. In fact, the use of fresh and living plant material is beneficial to visualize underlying physiological processes such as water flow (e.g., [123]). Some MRIs allow plants to grow over a period of several weeks in specially designed climate chambers [123]. This potentially allows to directly observe flower development in vivo and in real time. Nevertheless, surprisingly few flower developmental studies using MRI were conducted [124,125], and none of these studies took advantage of the real-time visualization aspect. Also, the aspect of mechanical forces was so far not addressed. The clear advantage is that not only the outcome of mechanical forces can be studied in 2D or 3D, but also the development itself. Primordia and organs that make contact to each other can theoretically be observed over their full development, allowing to test hypotheses on organ interaction so far only indirectly addressed. Apart from the mechanical process of development, also phytochemical observations can be made, and water flow can be monitored. This allows for a direct link between turgor pressure as an important factor in flower development [126]. These opportunities of directly observing flower development and linking development with physiological processes has immense potential for the study of the role of mechanical forces in flower development.

Overall, 3D techniques that allow to visualize intact flowers in different developmental stages helps to revolutionize the study of mechanical forces in flower development.

## 5. Conclusions and Outcomes

Mechanical forces are an intrinsic part of flower development and can be understood as required factors in developing the floral phenotype. Most of these influences take place at an early stage of development when meristems can be molded by compressing structures such as bracts, axes, or an outer perianth. Sectors of the floral bud experiencing higher contact pressure would then experience inhibition or retardation of organ initiation and growth, with petals and stamens usually fluctuating in response as sacrificed organs. Where and when the contact pressure occurs is species specific and depends on the very internal architecture and configuration of the bud. Pressures also confer a specific geometry to the meristematic tissues, codetermining the spatial distribution of organs or flowers that initiate on it, and even influence their blooming sequence. The findings herein presented indicate that these forces may be the trigger for specific developmental genetic mechanisms, as an epigenetic factor. Alternatively, these mechanical events may be integrated in the plant organism and autonomously repeated generation after generation. Eventually this internal environment could trigger a constant genetic mechanism that persists even when the initial force disappears. As discussed by Karpunina et al. [61] and El et al. [127], it remains difficult to test whether occurring changes are the effect of mechanical pressure or a prepatterning process affecting the organs and their shape. However, one could argue that prepatterning is a mechanical force exercised at the primordial level, determining the fate and position of organs before they are visible.

We thus highlight the generative role of mechanical forces in shaping the floral phenotype and underline our general view of flower development as the sum of interactions of known physiological and genetic processes, together with physical aspects and mechanical events that are entangled toward the shaping of the mature flower. The influence of mechanical forces in late developmental stages of flowers (e.g., forces related to pollination) will be addressed in future works.

This understanding of floral development offers a theoretical framework, and it also stimulates the conjecture of developmental hypotheses that can be tested by observational means on the plant intimate architecture and configuration using traditional and modern techniques in the study of the floral form.
